# Cells in the polyaneuploid cancer cell (PACC) state have increased metastatic potential

**DOI:** 10.1007/s10585-023-10216-8

**Published:** 2023-06-16

**Authors:** Mikaela M. Mallin, Nicholas Kim, Mohammad Ikbal Choudhury, Se Jong Lee, Steven S. An, Sean X. Sun, Konstantinos Konstantopoulos, Kenneth J. Pienta, Sarah R. Amend

**Affiliations:** 1 Cellular and Molecular Medicine Graduate Training Program, Johns Hopkins School of Medicine, Baltimore, MD USA; 2 Cancer Ecology Center, James Buchanan Brady Urological Institute, Johns Hopkins Medical Institute, Baltimore, MD USA; 3 Rutgers Institute for Translational Medicine and Science, New Brunswick, NJ USA; 4 Department of Mechanical Engineering, Johns Hopkins University, Baltimore, MD USA; 5 Department of Chemical and Biomolecular Engineering, Johns Hopkins University, Baltimore, MD USA

**Keywords:** Polyaneuploid cancer cells (PACCs), Polyploid giant cancer cells (PGCCs), Motility, Chemotaxis, Deformability, Vimentin

## Abstract

Although metastasis is the leading cause of cancer deaths, it is quite rare at the cellular level. Only a rare subset of cancer cells (~ 1 in 1.5 billion) can complete the entire metastatic cascade: invasion, intravasation, survival in the circulation, extravasation, and colonization (i.e. are metastasis competent). We propose that cells engaging a Polyaneuploid Cancer Cell (PACC) phenotype are metastasis competent. Cells in the PACC state are enlarged, endocycling (i.e. non-dividing) cells with increased genomic content that form in response to stress. Single-cell tracking using time lapse microscopy reveals that PACC state cells have increased motility. Additionally, cells in the PACC state exhibit increased capacity for environment-sensing and directional migration in chemotactic environments, predicting successful invasion. Magnetic Twisting Cytometry and Atomic Force Microscopy reveal that cells in the PACC state display hyper-elastic properties like increased peripheral deformability and maintained peri-nuclear cortical integrity that predict successful intravasation and extravasation. Furthermore, four orthogonal methods reveal that cells in the PACC state have increased expression of vimentin, a hyper-elastic biomolecule known to modulate biomechanical properties and induce mesenchymal-like motility. Taken together, these data indicate that cells in the PACC state have increased metastatic potential and are worthy of further in vivo analysis.

**Supplementary Information:**

The online version contains supplementary material available at 10.1007/s10585-023-10216-8.

## Introduction

Metastasis describes the development of secondary malignant growths at a distant site and is responsible for 90% of cancer related deaths [1]. The metastatic cascade describes the path of cancer cells as they travel through the body: invasion, intravasation, survival in the circulation, extravasation, and colonization. To form lethal metastatic lesions, cancer cells in the primary tumor must first acquire an invasive capacity to penetrate surrounding extracellular matrices. Second, the cells must enter the circulatory system via a process called intravasation, extending into neighboring vascular tissues and maneuvering between endothelial cells. This requires altered cancer cell cytoskeletal dynamics that increase deformability yet simultaneously preserve nuclear envelope integrity. Once in the circulatory system, the cells, now called circulating tumor cells (CTCs), must (1) evade anoikis, a form of programmed cell death initiated upon detachment from extracellular matrix and (2) survive the shear stress of blood flow. Fourth, the cells must extravasate out of the circulatory system into distant secondary organs. Lastly, the cells, now called disseminated tumor cells (DTCs), must survive and subsequently proliferate to form an overt metastatic lesion [2]. A cancer cell must be capable of successfully completing all five steps of the metastatic cascade to create a clinically-detectable metastasis.

Although metastasis is common at the organismal level (roughly 30% of cancer patients will develop metastases) [1], clinically-based mathematical modeling predicts that at the cellular level, metastasis is actually an extremely rare event. Estimates based on (1) the number of CTCs detectable in a patient’s blood, (2) the number of DTCs detected in their bone marrow (a common metastatic site for multiple cancers), and (3) the number of clinically detectable metastatic lesions suggest that only 1 of every 1.5 billion CTCs successfully completes the metastatic cascade [3]. Note that these calculations exclude cancer cells that never acquire motility; ergo, the proportion of metastasis-competent cells in the primary tumor is even lower. The identity of the rare subpopulation of cancer cells with true metastatic competency remains an open research question.

Conventionally, it is understood that cellular stress activates a canonical stress response, resulting in a brief pause in cell cycle (during which damage is repaired) followed by either swift cell cycle reactivation, apoptosis, or indefinite persistence in a non-cycling state [4–6]. The Polyaneuploid Cancer Cell (PACC) state describes a recently appreciated novel stress-response fate [7–16]. The PACC state is a transient, adaptive state adopted by some cells in response to stress [17]. The PACC state is characterized by physically enlarged cell size, increased genomic content, and lack of cell division, all of which can be attributed to endoreduplication [14, 18–20]. Endoreduplication is a common cell cycle variant in which the nuclear genome is replicated in the absence of mitosis, leading to polyploidy. Cells in the PACC state (PACCs) have been described using other names, including Polyploid Giant Cancer Cells, Multinucleated Giant Cancer Cells, and Pleomorphic Cancer Cells, among others [21–24]. Here, polyaneuploid specifically describes the stress-induced polyploidization of an pre-existing aneuploid cancer cell genome. Regardless of nomenclature, these types of cells have repeatedly been implicated in metastasis and chemotherapeutic resistance [19, 25–42].

The PACC state is accessed in response to many stressors, including hypoxia, acidity, ionizing radiation, and various classes of chemotherapeutic drugs, including cisplatin, docetaxel, and etoposide [7, 43]. Moreover, the PACC state has been described in multiple cancer types, including prostate cancer lines PC3, DU145, and LNCaP, breast cancer line MDA-MB-231, and ovarian cancer cell lines HEY and SKOv3, among many others [29]. Cells in the PACC state can be found at low numbers in untreated cultures, likely reflecting the low but ever-present cellular stress inherent to routine cell culture practices. Application of additional tumor microenvironmental stressors causes a marked increase in the number of PACCs. While some of the cells exposed to the applied stress engage in canonical stress-response programs resulting in brief cell cycle pauses, apoptosis, and cell-cycle exit, a subset of cells exposed to the applied stress initiate a Polyaneuploid Transition (PAT) to access the endoreduplicative PACC state [44]. Upon accession of the PACC state, cells retain high levels of cellular functionality, including operative respiration, biosynthesis, digestion, absorption, secretion, homeostasis, transport, and movement [45]. Eventually, cells undergo ploidy-reductive depolyploidization and exit the PACC state by engaging reductive cell division to produce proliferative non-PACC progeny of normal physical size and typical genomic content [43, 46–54].

Cells in the PACC state have been identified histologically in various human cancer types in both primary and metastatic lesions. Presence of cells in the PACC state within primary tumors is generally associated with poorer prognosis [55, 56]. Recently, it was shown that the presence of PACC state cells in the primary tumors of men with prostate cancer who underwent radical prostatectomy with curative intent is predictive of lower metastasis-free survival [57, 58]. Additionally, rapid autopsy of 5 men with metastatic prostate cancer revealed presence of cells in the PACC state in all distant metastatic lesions analyzed [59]. In animal models, highly metastatic prostate cancer cells selected by serial metastatic passage in mice are highly enriched for cells in the PACC state [60]. Taken together, these data suggest that cells in the PACC state may have increased metastatic potential. This hypothesis predicts that PACC state cells can successfully invade, in/extravasate, and survive in the circulation. To test each of these predictions, we respectively quantified the motility, deformability, and liquid-environment survivability of cisplatin-induced PACCs derived from the PC3 prostate cancer cell line.

## Results

### Cancer cells undergo a polyaneuploid transition (PAT) in response to chemotherapeutic stress

**Fig. 1:**
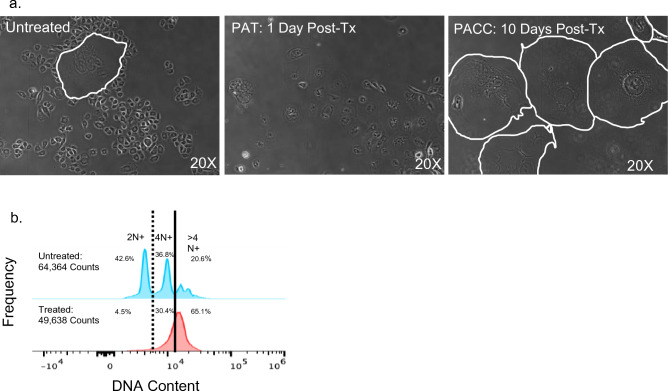
Cancer cells undergo a Polyaneuploid Transition in response to applied stress: **a** 2D morphology of PACC state induction in PC3 cells using a 72 h dose of 6 μM cisplatin. Cell populations are untreated, undergoing a PAT 1 day post-treatment, or have entered a definitive PACC state 10 days post-treatment. Acquired by 20X phase microscopy. PACC cell borders are demarcated in white. **b** Relative DNA content of untreated cells vs. treated cells 1 day post-treatment. Acquired by flow cytometry

The PC3 prostate cancer cell line was treated with 6 μM of cisplatin for 72 h, after which cisplatin-containing media was replaced with fresh media. After treatment, all cells had characteristics of PACC morphology (increased cell size, increased nuclear size, distinctive nuclear morphology, and increased peri-nuclear granularity including increased lipid droplet content), indicating they had initiated PAT (Fig. 1a). Polyploidization was validated by flow cytometry on a subset of cells to measure the relative DNA content of treated cells compared to untreated controls. The cell cycle plots of treated cells demonstrate a clear rightward shift compared to the typical cell cycle plot of untreated controls, indicating a > G2 ploidy (Fig. 1b). All remaining treated cells were maintained in culture and monitored for an additional 10 days via in-incubator time lapse microscopy. Treated cells did not undergo any cellular division during this time, but they did continue to increase in size (i.e. 2D surface area) throughout the duration of the experiment. Treated cultures continued to show evidence of cell death until day 10 after treatment release, after which all surviving cells are definitively in the PACC state (Supplemental Fig. 1). All following experiments were completed with PACCs maintained in culture without dividing for 10 days post cisplatin-treatment, unless otherwise indicated.

### Cells in the PACC state have increased motility

Motility is critical for metastasis initiation and is a prerequisite to the first step of the metastatic cascade. To directly quantify the motility of cells in the PACC state compared to non-PACC parental cells, we performed time lapse microscopy followed by single cell tracking using ImageJ. Motility videos of non-PACCs and PACC state cells are included as Online Resource 1 and 2, respectively. All cells were exposed to uniform 20% FBS-supplemented media conditions, denoted as + / + .

**Fig. 2:**
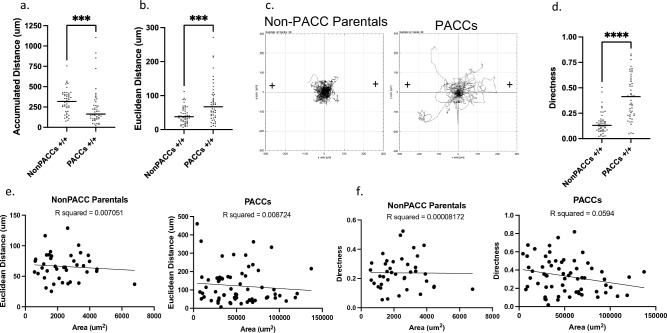
Cells in the PACC state are more motile: **a**, **b** Quantification of the (**A**) accumulated distance and (**B**) Euclidean distance travelled by non-PACC parental cells or PACCs in uniform 20% FBS-supplemented media conditions (+ / +) throughout a 24-h time lapse. **c** Spider plots depicting the motility tracks of single non-PACC parental cells (n = 50) or PACCs (n = 49) in uniform 20% FBS-supplemented media conditions (+ / +) throughout a 24-h time lapse. **d** Directness of travel demonstrated by non-PACC parental cells or PACCs in uniform 20% FBS-supplemented media conditions (+ / +) throughout a 24-h time lapse. **e** Linear regression comparing the Euclidean distance travelled and 2D cell surface area of non-PACC parental cells and PACCs. **f** Linear regression comparing the Directness and 2D cell surface area of non-PACC parental cells and PACCs

While cells in the PACC state travelled lesser accumulated distances (p value = 0.0002) than non-PACC parental cells (Fig. 2a), cells in the PACC state travelled greater Euclidean distances (p value = 0.0002) than non-PACC parental cells (Fig. 2b). Accumulated distance describes the total distance traveled by a cell, and Euclidean describes the net distance traveled by a cell (i.e., the length of the line connecting the starting and ending points). Spider plots tracking the (X,Y) coordinates of each cell’s trajectory mapped on a Cartesian plane wherein all cell locations at Time 0 are translated to the origin (0,0) reveal that the observed increase in accumulated distance travelled by non-PACC cells is almost entirely non-migratory: the cells’ motility is mostly in place – no large net distances are travelled (Fig. 2c). Thus, Euclidean distance is a more metastasis-relevant measure of invasive potential than accumulated distance. The ratio of Euclidean distance over accumulated distance can be used to calculate directness of movement. A directness metric of 1 describes movement in a perfectly direct (i.e. straight) line, while a directness metric approaching 0 indicates totally indirect cell movement. In our analyses, cells in the PACC state moved more directly (p value < 0.0001) than non-PACC parental cells (Fig. 2d).

To test if increased PACC motility is attributable to the increased cell size of the PACC state population, we performed a linear regression comparing the 2D surface area of each cell to its specific motility metrics. No correlation between cell size and cell motility within non-PACC parental cell populations or within PACC state populations was found (Fig. 2e). All linear regressions returned nonsignificant R^2^ values when modeling both the relationship between cellular area and Euclidean distance for both non-PACC parental cells (R squared = 0.007051) and cells in the PACC state (R squared = 0.008724) as well as the relationship between cellular area and directness for both non-PACC parental cells (R squared = 0.00008172) and PACC state cells (R squared = 0.0594) (Fig. 2e and 2f).

### Cells in the PACC state demonstrate a directional response to a chemotactic FBS gradient

A motile cell’s speed (ergo, distance travelled) and directness are considered its kineses, a term borrowed from foraging and movement ecology used to describe basic movement parameters such as kinesis, klinokenesis or orthokinesis [61]. Cellular movement is often kineses-directed, meaning changes in a cell’s location are directly due to cell-intrinsic adjustments to its speed and/or direction, rather than due to some extrinsic applied force. Kineses-directed movement occurs in response to local resource levels, such as directional chemotaxis up a nutrient gradient [62].

**Fig. 3:**
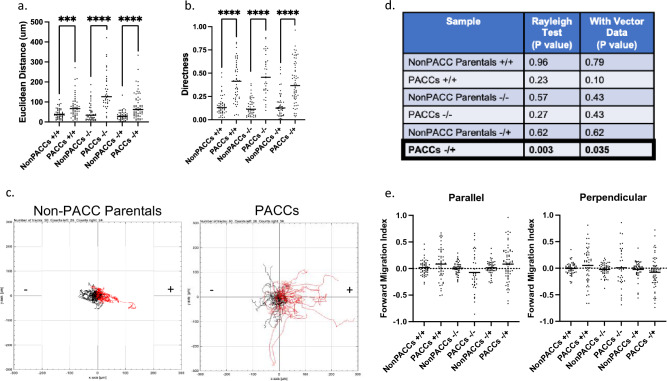
Cells in the PACC state demonstrate a directional response to a chemotactic FBS gradient: **a**, **b** Quantification of the (**A**) Euclidean distance travelled by or (**B**) directness of movement of non-PACC parental cells or PACCs in either uniform 20% FBS-supplemented media conditions (+ / +), uniform serum-free media conditions (−/−), or a chemotactic gradient of 0–20% FBS-supplemented media (−/ +) throughout a 24-h time lapse. **c** Spider plots depicting the motility tracks of single non-PACC parental cells (n = 50) or PACCs (n = 60) in a chemotactic gradient of 0–20% FBS-supplemented media (−/ +) throughout a 24-h time lapse. Red tracks indicate cells that had a net migration toward up the FBS gradient. **d** Results of the Rayleigh Test and Rayleigh Test with Vector Data for approximation of chemotactic behavior in non-PACC parental cells and PACCs. **e** Parallel (to FBS gradient) and Perpendicular (to FBS gradient) Forward Migration Indices for assessment of chemotactic behavior/chemotaxis in non-PACC parental cells and PACCs

To test if cells in the PACC state are capable of resource sensing (i.e., perform kineses-directed movement or directional chemotaxis), we performed time lapse microscopy and single cell tracking along an FBS gradient of 0–20% FBS (−/ +). Positive control cells were cultured in uniform 20% FBS conditions (+ / +) and negative control cells were cultured in uniform serum-free conditions (−/−). As we had previously observed, cells in the PACC state always moved greater Euclidean distances than non-PACC parental cells under all experimental conditions (+ / + p value = 0.0002, −/− p value < 0.0001, −/ + p value < 0.0001) (Fig. 3a). Furthermore, cellular migrations were more direct (i.e., persistent) in PACCs than non-PACC parental cells under all experimental conditions (+ / + p value < 0.0001, −/− p value < 0.0001, −/ + p value < 0.0001) (Fig. 3b). Spider plots tracking the (X, Y) coordinates of each cell’s trajectory indicate that cells in the PACC state respond more robustly to presence of an FBS gradient than non-PACC parental cells. (Fig. 3c).

To assess if cells in the PACC state exhibited directional chemotaxis, we performed the Rayleigh Test and the Test of Forward Migration Indices. The Rayleigh Test is a statistical test for the uniformity of circularly distributed data (here, the spider plots’ final X, Y coordinate of a cell’s trajectory) [63]. The null hypothesis is circular uniformity. Final coordinates distributed equally around the origin indicate lack of directional response to the FBS gradient and will fail to reject the null, producing a nonsignificant p value. Final coordinates distributed nonequally around the origin indicate presence of a directional response and will reject the null, producing a significant p value. The Rayleigh Test for vector data also includes a parameter of distance, measured between the final coordinate and the origin. To define the movement of cells as chemotactic, the result of the Rayleigh tests for the gradient condition must be significant while the results for the control conditions must be nonsignificant. Both the Rayleigh Test and the Rayleigh Test for vector data were only significant for PACC state movement under gradient conditions (Fig. 3d). This indicates that cells in the PACC state exhibited directional chemotaxis in response to an FBS gradient but that non-PACC parental cells did not.

The Test of Forward Migration Indices quantifies the directional efficiency of cellular movement in a defined direction. The Parallel Forward Migration Index ( || FMI) describes the movements of cells in planes parallel to that of a present gradient. Positive || FMI values represent “forward” movement up the gradient, while negative || FMI values represent “backwards” movement down the gradient. The Perpendicular Forward Migration Index (⊥ FMI) describes the movements of cells in planes perpendicular to that of a present gradient. To define the movement of cells as chemotactic, i) the || FMI of the gradient condition must be significantly higher than the || FMI of both control conditions, ii) the || FMI of the gradient condition must be significantly higher than the ⊥ FMI of the gradient condition, which must be close to zero, and iii) the || FMI and ⊥ FMI of both control conditions must be close to zero. Our data indicate that directional chemotaxis in response to an FBS gradient is apparent in PACCs but not in non-PACC parental cells (Fig. 3e).

### Cells in the PACC state have altered cytoskeletal stiffness

The second step of the metastatic cascade, intravasation, requires substantial morphological deformation as invasive cells maneuver between endothelial cell junctions. Cytoskeletal stiffness is an important cellular property that changes during migration and can be used as a proxy measure of deformability. Cytoskeletal stiffness can be described and measured in two ways: intracellular network stiffness and cortical stiffness [64].

**Fig. 4:**
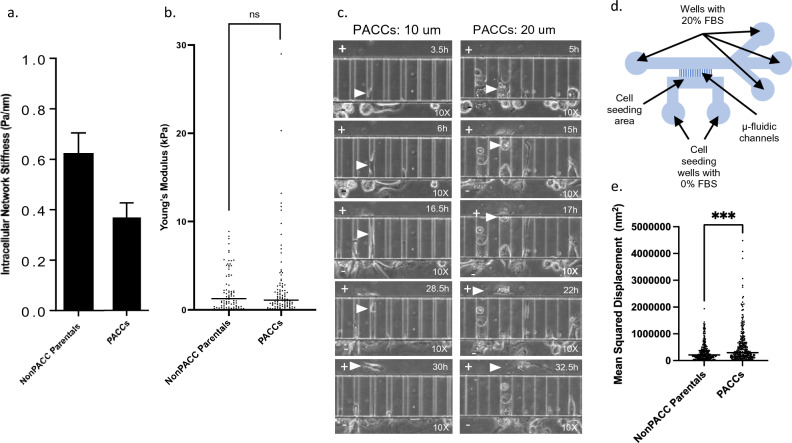
Cells in the PACC state have altered cytoskeletal stiffness and increased cytoskeletal dynamics: **a** Intracellular network cytoskeletal stiffness of non-PACC parental cells PACCs acquired via MTC using ferromagnetic RGD-linked beads. **b** Young’s Modulus (or cortical stiffness) of the peri-nuclear region of non-PACC parental cells and PACCs acquired using AFM. **c** Demonstration of functional deformability in PACCs: Evidence of simultaneous migration and deformation of PACCs through 10 μm and 20 μm wide channels of a custom invasion channel microfluidic device throughout a 36-h time lapse. **d** Schematic of the microfluidic device. **e** Mean Squared Displacement of spontaneous movement of RGD-linked beads attached to the cytoskeleton of non-PACC parental cells and PACCs throughout a 5-min time lapse

Magnetic Twisting Cytometry (MTC) was used to measure the intracellular network stiffness. Using MTC, cells in the PACC state showed decreased intracellular network stiffness compared to non-PACC parental cells. This result indicates that cells in the PACC state are more deformable than non-PACC parental cells (Fig. 4a).

Atomic Force Microscopy (AFM) was utilized as an orthogonal approach to further evaluate cortical stiffness in the perinuclear region of cells. Cortical stiffness is reported as Young’s modulus (E, or also elastic modulus), where a larger Young’s modulus indicates a stiffer, or less deformable, cell. By AFM there was no significant difference between the perinuclear Young’s modulus (i.e. cortical stiffness) of cells in the PACC state and that of non-PACC parental cells (p value = 0.5765) (Fig. 4b).

Given the discrepancies between our MTC and AFM data, we sought to investigate the functional deformability of cells in the PACC state. To directly assess functional deformability, we simultaneously tracked cell movement and deformation through a custom-built microfluidic invasion chamber [65, 66]. In this device, cells are seeded in a primary chamber that is connected to a secondary chamber via narrow invasion channels of varying sizes (3, 6, 10, 20, and 50 μm). Cells invade from the primary chamber to the secondary chamber by squeezing through the invasion channels (Fig. 4d). A 0–20% FBS gradient was applied across the invasion channels to stimulate directional movement. Time lapse imaging demonstrated that cells in the PACC state are capable of functional deformability; PACC state deformed while simultaneously moving into and through the 10 μm and 20 μm invasion channels (Fig. 4c). Videos of PACC functional deformability through 10 μm and 20 μm channels are included as Online Resources 3 and 4, respectively.

### Cells in the PACC state exhibit increased cytoskeletal dynamics

The cytoskeleton is the primary organelle responsible for cellular migration and deformability. It has been reported that increased measures of cytoskeletal dynamics correlate with increased cellular migration and deformability [67]. Cytoskeletal dynamics were evaluated by functionalizing RGD-coated microbeads to the cells’ cytoskeleton through cell surface integrin receptors and tracking individual bead displacement via short-range time lapse microscopy. Spontaneous bead motions report the underlying remodeling dynamics of the cytoskeletal network to which the beads are attached (i.e., greater cytoskeletal rearrangement dynamics are reflected as greater RGD bead displacement, reported as mean squared displacement). Cells in the PACC state had significantly greater RGD bead displacement than non-PACC parental cells (p value = 0.0002), indicating that cells in the PACC state rearrange their cytoskeleton more dynamically (Fig. 4e). This data is consistent with the direct observations of increased motility and increased deformability in the PACC state.

### Cells in the PACC state have increased vimentin expression

Vimentin (VIM) is a Type III Intermediate filament component of the cytoskeleton and a canonical marker of Epithelial-to-Mesenchymal transition (EMT). VIM has been implicated in two major cellular functions: mesenchymal cell motility and preservation of nuclear-cytoskeletal integrity, especially in response to compressive stress. It has also been found to play an important role in cell spreading on firm 2D substrates [68–76].

**Fig. 5:**
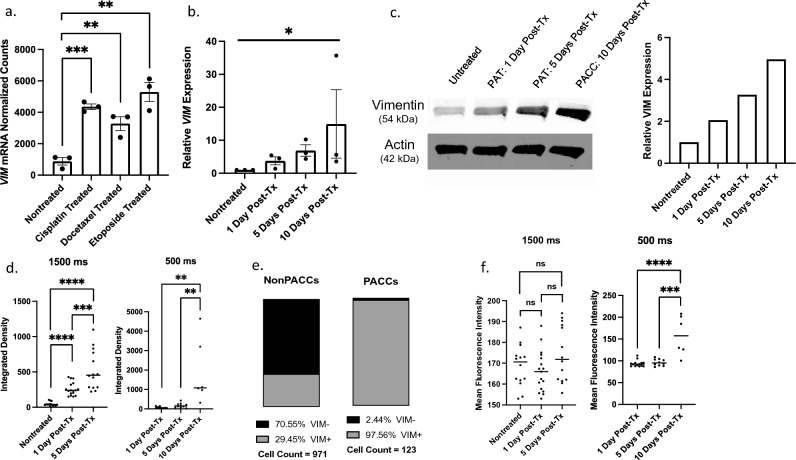
PACCs have increased expression of vimentin at both the RNA and Protein level: **a** mRNA Nanostring analysis comparing normalized counts of *VIM* mRNA transcripts in untreated, cisplatin-treated, docetaxel-treated, and etoposide-treated populations. **b** RT-qPCR analysis of relative *VIM* expression between untreated cells, cells undergoing a PAT 1 day post-treatment, cells undergoing a PAT 5 days post-treatment, and PACC cells 10 days post-treatment. **c** Western blot and quantitative densitometry of VIM expression in untreated cells, cells undergoing a PAT 1 day post-treatment, cells undergoing a PAT 5 days post-treatment, and PACC cells 10 days post-treatment. **d** Integrated density of single-cell VIM signal in untreated cells, cells undergoing a PAT 1 day post-treatment, cells undergoing a PAT 5 days post-treatment, and PACC cells 10 days post-treatment using either a 1500 ms exposure time or a 500 ms exposure time. **e** Percentage of VIM positive cells by immunofluorescence tiled imaging in non-PACC parental and PACC populations. **f** Mean Fluorescent Intensity of single-cell VIM signal in untreated cells, cells undergoing a PAT 1 day post-treatment, cells undergoing a PAT 5 days post-treatment, and PACC cells 10 days post-treatment using either a 1500 ms exposure time or a 500 ms exposure time

We assessed VIM levels at both the RNA and protein level in cisplatin-induced cells at various time points throughout the PAT as well as in cells in a definitive PACC state. mRNA NanoString analysis of treated and untreated cells immediately following 72-h exposure to IC50 doses of either cisplatin, docetaxel, or etoposide showed a 3.5–fivefold increase in *VIM* mRNA upon entry into the PAT (Fig. 5a). This was validated by RT-qPCR analysis: cisplatin-treated cells had increased *VIM* expression compared to untreated controls. *VIM* expression steadily increased with time post-treatment throughout the PAT and into the definitive PACC state (10 days post-treatment) (Fig. 5b). At the protein level, western blot analysis of treated and untreated cells likewise showed that VIM protein expression increased with cisplatin-induction and continued to steadily increase with time post-treatment throughout the PAT and into the definitive PACC state (10 days post-treatment) (Figs. 5c).

Immunofluorescent staining was used to assess VIM expression at the single-cell level. Total per-cell VIM expression as determined by Integrated Density (i.e. summation of fluorescence intensity in all pixels of a defined cell) was significantly higher in cisplatin-induced cells than in untreated controls, and continued to steadily increase with time post-treatment throughout the PAT and into the definitive PACC state (10 days post-treatment) (p value = 0.0360) (Fig. 5d), recapitulating the data obtained via western blot. Of note, the difference in VIM staining intensity between treated cells 10 days post-treatment (PACC state) and untreated controls (non-PACC parental cells) was appreciably different between the two groups, limiting the visualization and imaging with the same exposure time. For example, an exposure time that is sufficient to visualize VIM + signal in untreated controls (1500 ms) was too long for treated cells 10 days post-treatment, resulting in an oversaturated and unanalyzable image. Accordingly, a reduction in exposure time to achieve an appropriately saturated image of the treated cells 10 days post-treatment (500 ms) renders the signal of the untreated controls invisible (Supplemental Fig. 2). Although these intensity differences between the two groups limited their direct comparability, it was apparent that treated cells undergoing the PAT and entering the PACC state showed markedly increased VIM expression.

Quantification of VIM positivity of cells in the PACC state and non-PACC parental cells showed that 98% of cells in the PACC state were VIM + (120/123) compared to 29% (286/971) of non-PACC parental cells (Fig. 5e). The localization pattern of VIM within VIM + cells also differed between PACCs and non-PACC parental cells. In non-PACC parental cells, VIM staining showed intensely bright and punctate or clustered peri-nuclear signal. In contract, VIM staining showed more dim and diffuse filamentous networks of fibers spanning the entire cytoplasm in PACCs. In addition to visual approximations, this localization difference can also be quantified by the Mean Fluorescent Intensity (MFI, or average fluorescence intensity per pixel among all pixels of a defined cell). Again, images were taken and analyzed using two different exposure times (1500 ms and 500 ms) to accurately capture both untreated cells and treated cells 10 days post-treatment. Specifically, MFI quantifications showed no significant differences between untreated cells and two groups of treated cells in various stages of the PAT analyzed after various number of days post-treatment (Fig. 5f). Treated cells analyzed 10 days post treatment (PACC state) did have an increased MFI compared to untreated (non-PACC parental) cells, indicating that VIM overexpression dominates VIM content dynamics more so than VIM redistribution or re-localization does by 10 days post-treatment (Fig. 5f). These VIM localization findings match that of other recently published work [77].

### Increased VIM partially explains increased PACC state motility

**Fig. 6:**
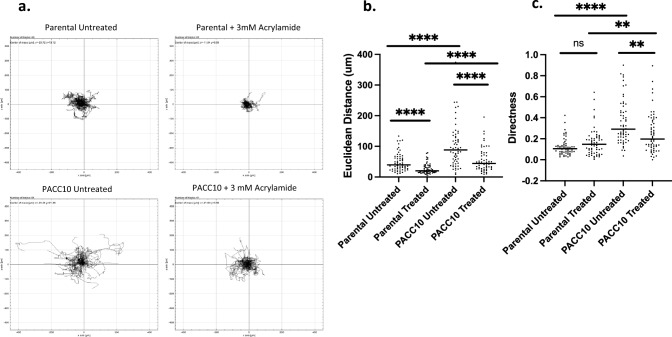
Vimentin ablation causes decreased motility in PACCs: **a** Spider plots depicting the motility tracks of single untreated non-PACC parental cells (n = 60), 3 mM acrylamide pre-treated non-PACC parental cells (n = 61), untreated PACCs (n = 61), or 3 mM acrylamide pre-treated PACCs (n = 61) throughout a 24-h time lapse. **b**, **c** Quantification of the b) Euclidean distance and c) directness travelled by untreated or treated non-PACC parental cells and untreated or treated PACCs throughout a 24-h time lapse

To assess the role of VIM in PACC motility, we pre-treated non-PACC parental cells and PACCs with acrylamide to ablate VIM polymerization via destabilization and then performed a time lapse motility experiment, as in Fig. 2a. The dose was selected based on the results of an acrylamide dose response curve’s effects on the stability of three major cytoskeletal components VIM, pan-tubulin and beta-actin, as measured by immunofluorescent imaging (Supplemental Fig. 3a–b). We compared the Euclidean distance travelled and directness of cell movement of 3 mM acrylamide treated and non-treated cells and found that acrylamide treatment reduced the Euclidean distance travelled of both non-PACC parental cells and PACCs and reduced the directness of PACC motility (Fig. 6a-c).

To assess the independence of VIM in PACC motility, we performed a chemotactic migration experiment identical to that in Fig. 3, and then fixed and performed IF for VIM protein immediately following the final captured time lapse image. We compared end-point VIM expression and motility parameters of PACCs by linear regression. There was no correlation between measures of VIM Integrated Density (i.e. total VIM content per cell) and any motility parameter analyzed, including Euclidean distance travelled (R squared = 0.0009918), directness of PACC movement (R squared = 0.007451), or accumulated distance travelled (R squared = 0.0005488). Similarly, there was no correlation between the natural log of VIM MFI (i.e. surrogate approximate of VIM localization) and any motility parameter analyzed. (Euclidean distance R squared = 0.01333) (Directness R squared = 0.005681) (Accumulated distance R squared = 0.04932) (Supplemental Fig. 4a–f).

### Cells in the PACC state are equally as anoikis resistant as non-PACC parental cells

Following invasion and intravasation, a cell is exposed to multiple metastatic barriers in the circulatory system. Among these barriers is anoikis: a form of programmed cell death signaled by ECM detachment. To test the anoikis resistance of PACCs and non-PACC parental cells, we used a serial low-adhesion tissue culture plate to normal-adhesion tissue culture plate re-adhesion assay followed by metabolic cell viability quantification. We’ve observed that PC3 cells maintain this level of anoikis resistance as they undergo PAT to enter the PACC state: Cells in the PACC state were equally as anoikis resistant as non-PACC parental cells (Supplemental Fig. 5a). By utilizing a selection-free CTC platform and modified analysis to include polyploid cancer cells, anecdotal evidence of cells in the PACC state (as defined by increased nuclear content, negative for hematopoietic markers, positive for epithelial markers, and increased VIM content) in the circulation of a patient with metastatic castrate-resistant prostate cancer (mCRPC) was identified (Supplemental Fig. 5b).

## Discussion

There are barriers to successful metastasis at every step of the metastatic cascade. Only metastasis-competent cells can successfully invade the primary organ, intravasate into the circulation, survive, extravasate into a distant secondary site, and then colonize there to form a clinically detectable micrometastatic lesion. Identification of the rare subtype of completely metastasis-competent cancer cells remains one of the most important goals of cancer research.

Cells in the PACC state represent a subset of cells that have engaged an evolutionarily conserved and developmentally-deployed polyploidization program in response to environmental stress. The PACC state is characterized by endoreduplication: an atypical cell cycle variant that drives increased genomic content, increased physical size, and lack of mitotic cell division. After sufficient stress-recovery, cells re-engage a canonical cell cycle, undergoing ploidy-reductive cell division to create proliferative cancer cells. Endoreplication and eventual depolyploidization make cells in the PACC state distinct from cells experiencing classically defined senescence, in which cells exhibit total and permanent cell cycle arrest. We do not observe the indefinite G1 or G0 arrest typically associated with canonical senescence. After a period of recovery, endoreplicative cells in the PACC state reenter a proliferative state, further distinguishing them from the terminal state of classically defined senescence [20]. Of note, the term “senescent” now describes a highly heterogenous population presenting with variable phenotypes. Indeed, multiple variants of senescence (replicative, oxidative, oncogene-induced, therapy-induced, chromosomal-instability-induced, etc.) demonstrate unique cell life cycles distinct from classically defined irreversible senescence. Indeed, morphological similarities between cells in the PACC state and reports of cells that have undergone therapy-induced senescence, and indeed some recent work has described a potential link between therapy-induced senescence and polyploidy or endocycling [78, 79]. Furthermore, work on chromosomal-instability-induced senescence demonstrates that senescent tetraploid cells contributed to increased tumorigenic and metastatic potential [80, 81]. For an endocycling PACC/PGCC-state cell and some flavor of non-canonical-senescence-associated cancer cell to be two names for an identical population hinges on the literature actively working to define reversible and/or polyploid-associated varients of senescence in the context of cancer [82–84].

Several features of the PACC state indicate this cell phenotype may play a key role in metastasis. Previously, cells in the PACC state have been detected in patient primary tumors and metastatic lesions. Additionally, other published work outside of the PGCC field has identified increased ploidy as contributing to metastatic potential [80]. Here, we show that PACC state cells demonstrate kineses-directed motility and deformability, factors that predict successful invasion and intravasation/extravasation. Furthermore, the transience of the PACC state predicts successful distant site colonization following PACC depolyploidization. Additionally, we also present a potential role for the cytoskeletal filament vimentin in driving these metastasis relevant PACC phenotypes.

Specifically, we conclude that cells in the PACC state are more motile than non-PACC parental cells. The large and dynamic size of cells in the PACC state limits the use of traditional Boyden chamber assays and scratch-wound assays to measure motility. We used single-cell tracking following time lapse microscopy to measure multiple movement parameters, including net Euclidean distance travelled and directness of movement. Euclidean distance is a more metastasis-relevant measure of invasive potential than accumulated distance; it captures net distances travelled away from primary tumors (a cancer cell’s initial location) that cannot be captured by measures of accumulated distance. Other groups studying PACC motility in an adjacent cancer cell models, including cells line other than PC3, have also shown that cells in the PACC state demonstrate increased motility and increased persistence (i.e., an alternative measure of directness) compared to non-PACC parental cells [77]. Though it is conceivable that the increased size of cells in the PACC state could cause a proportional increase in their “step size,” we find that any such scaling factor cannot explain the observed increases in PACC motility: there is no correlation between PACC state cell area and PACC motility parameters. Rather, this suggests cancer cells activate specific motility programs as they undergo PAT in response to applied stress.

In addition to quantifying motility, we also performed qualitative analyses of PACC state migration movies. Such analyses showed that cells in the PACC state primarily perform mesenchymal-type migration. In this movement type, the cells are flat and inch along using an alternating combination of pseudopodia elongation and trailing edge contraction. Cells in the PACC state with flat morphology can also move using a combination of smooth ruffling and gliding motions without obvious pseudopod formation or extension. Occasionally, clear ameboid-type migration can also be observed in PACC state cells, in which rounded-up cells bleb continuously and amorphously while traveling very quickly across the field of view [85, 86].

It is useful to study cancer cell motility in the context of the habitat selection theory of (organotropic) cancer metastasis [62]. Habitat selection uses both classic cancer and ecology modeling to emphasize the collateral importance of motility and environment sensing in metastasis-competence, positing that metastasis-competent cells engage in kineses-directed movement. This theory predicts that emigration beyond the primary tumor is driven by the resource-guided movements of cancer cells in search of resources. When considered in the context of the nutrient-depleted, hypoxic, and acidic tumor microenvironment, the ability to directionally respond to chemotactic resource gradients offers a clear adaptive advantage. Expressly, it asserts that invasion and intravasation/extravasation require both motility and resource-sensing capabilities. Application of the habitat selection model to our motility experimental framework revealed that cells in the PACC state directionally respond to a 0% to 20% FBS chemotactic gradient. For example, cells in the PACC state in uniform serum-free conditions traveled in nonspecific, unoriented directions. The addition of an FBS gradient re-oriented the direction of travel toward the higher FBS concentration while simultaneously preserving the increased net Euclidean distance traveled and directness of cells in the PACC state over non-PACC parental cells. This chemotactic ability appears to be gained as cells undergo PAT, as the directional movement of non-PACC parental cells is not influenced by the same gradient. Two statistical analyses of chemotaxis showed that cells in the PACC state exhibit directional chemotaxis in response to an FBS gradient but that non-PACC parental cells do not. As such, PACC state cells are either i) better able to sense the presence of an FBS gradient, ii) better adapted to directionally respond to one, or iii) both.

Interestingly, we found that cells in the PACC state in serum-free media conditions traveled the furthest net distance compared to PACC state cells in other FBS conditions. This difference was not observed in non-PACC parental cells. Similarly, PACC state cells in serum-free media conditions trended toward traveling the most directly compared to other FBS conditions, though this difference is not statistically significant. Again, this difference was not observed in non-PACC parental cells. These observations suggest that cells in the PACC state can sense and respond to environments devoid of nutrients by initiating direct movement (in any direction) away from their current nutrient-depleted location. This observation aligns with principles of optimal foraging theory, an ecological paradigm used to predict and describe how an organism behaves when searching for food. When no resources are available in an organism’s current location, it is most advantageous to pick a single direction and travel in it a long distance to increase the likelihood of encountering a new environment with adequate resources [62, 87, 88].

When a cell migrates, it experiences both tension and compression. The peripheral cytoplasmic region of a motile cell experiences tension (“pulling”) forces, particularly when employing mesenchymal-type migration. Simultaneously, the nucleus (the largest and least amorphous organelle in a cell), experiences compression (“pushing”) forces. Functional deformability is important during both invasion and intravasation/extravasation when motile cells experience both tension and compression as they maneuver through densely packed tumor cells, extra-cellular matrices, and endothelial cells [71]. Notably, nuclear integrity is the most important determinant of cellular viability in a cell experiencing deformation.

It is useful to study cancer cell deformability and nuclear integrity in the context of molecular biophysics, particularly using stress–strain diagrams. Stress–strain diagrams depict the causal relationship between applied forces (the stress) and resultant shape deformation (the strain) of an object. A hyper-elastic biomaterial is one with a nonlinear stress–strain relationship that undergoes extremely large deformations in response to minimal amounts of applied force. Hyper-elastic biomaterials are nearly incompressible, meaning they can change their shape while retaining near-constant volume. Furthermore, they readily return to their original shape when the force is removed. Most notably, hyper-elastic biomaterials stiffen dramatically when under intense compression, but exhibit softening when under tension [89].

Though the MTC data (showing cells in the PACC state have decreased stiffness) and AFM data (showing cells in the PACC state have equivalent or sometimes greater stiffness) initially appear to contradict one another, a molecular biophysical perspective reveals that together, they indicate that cells in the PACC state are hyper-elastic. In the MTC assay, a magnetic field exhibited a peripheral pulling force on the cytoskeleton, creating tension. During MTC, PACC state cells demonstrated decreased intracellular cytoskeletal network stiffness compared to non-PACC parental cells, which aligns with the characteristics of a hyper-elastic cytoskeleton experiencing tension. In the AFM assay, a pointed probe was depressed into the perinuclear region with uniform downward force, creating compression. During AFM, cells in the PACC state were on average equally as cortically stiff in the perinuclear region as non-PACC parental cells, though there did exist some cells in the PACC state with extremely stiff peri-nuclear regions far exceeding the stiffness of any non-PACC parental cell. The maintenance of (or occasional increase in) cortical stiffness in cells in the PACC state aligns with the characteristics of a hyper-elastic cytoskeleton experiencing compression. The hyper-elastic nature of the PACC state provides cells with both i) a greater capacity for invasion and intravasation-promoting peripheral deformability than non-PACC parental cells and ii) equal or greater nuclear integrity than non-PACC parental cells.

Previous published work shows that VIM intermediate filaments are hyper-elastic and thus allow cells, and particularly nuclei, to withstand extreme deformations without fracture or rupture [90]. Overall, this simultaneously confers both intracellular and cortical cytoskeletal strength and stretchability, in additional to the widely recognized role vimentin plays in conferring mesenchymal-like motility. In our model, all cells in the PACC state show increased *VIM*/VIM content at the RNA and protein level when compared to non-PACC parental cells by four orthogonal techniques, and ablation of this VIM content using low-dose acrylamide resulted in significantly decreased motility metrics. Irrespective of initial VIM levels, a subsequent decrease in VIM will decrease PACC state motility, indicating a necessary role for VIM in sustaining increased PACC state motility. Interestingly, treatment of non-PACC parental cells with acrylamide induced a PAT, indicating that while vimentin may be important for PACC state motility, it is not essential for PACC state entry. Though reduction of vimentin reliably produces a decrease in motility, vimentin is likely not the only driver of increased PACC state motility. Our observed lack of correlation between VIM abundance and PACC motility shows that VIM cannot independently explain the increased motility of cells in the PACC state.

Though one obvious limitation of this work is that it was performed in only the PC3 cell line, similar observations have been made by other scientists using other cell lines. Recent work published by Dawson et. al also showed that VIM content was increased in PACCs using a breast cancer MDAMB231 paclitaxel-induced PGCC (Polyploid Giant Cancer Cell, a term synonymous with PACC) model [77, 91, 92]. Dawson et. al. also showed that increased VIM content in their model is necessary for migratory persistence, or enhanced directness of PGCC motility. Chemical inhibition (with low-dose acrylamide) or siRNA-mediated knockdown of *VIM* resulted in both decreased PGCC spreading/surface area and decreased migratory persistence, supporting a role for vimentin in both cytoskeletal biomechanics and motility. Dawson et. al has also shown retained nuclear stiffness (i.e. integrity) in MDAMB231 paclitaxel-induced PGCCs [92]. Taken together, our work jointly suggests that VIM plays key roles in modulating the biophysical properties and orienting the polarization required for directed motility in PGCCs/PACCs.

To summarize, the observed motility, environment-sensing, and deformability of cells in the PACC state suggest increased invasion and intravasation potential. These phenotypic observations also predict increased extravasational competency in PACCs. The habitat selection model highlights the requirements of a CTC halted in the capillary of a distance secondary organ to i) sense presence of an appropriate resource within the tissue of the secondary organ and ii) directionally respond by travelling out of the circulation and into the secondary organ [62], during which it must deform as it moves between endothelial cells. Thus, the properties that predict a cell in the PACC state’s invasion and intravasation competency are the same ones that predict its extravasation competency.

While successful invasion, intravasation/extravasation are supportive of increased metastatic potential, a truly metastasis-competent cell must also be capable of survival in the circulation. Deformability is pertinent for survival in the circulation. CTCs must resist the shear stress of blood flow to survive the circulatory system long enough to reach capillary beds supplying a distant organ site. Increased deformability heightens a cell’s ability to withstand shear stresses of blood flow, as has been observed in studies of red blood cell dynamics [93]. Our observations of increased PACC state deformability indicate cells in the PACC state may be able to survive the shear stress of blood flow within the circulatory system. CTCs must also resist anoikis, a programmed cell death in response to cell:ECM detachment. Our observations of maintained PACC state anoikis-resistance indicate that accession of the PACC state does not decrease a PC3 cancer cell’s ability to survive in the circulation. Considering that the PC3 cell line was derived from an osseous metastatic lesion of a prostate cancer, it is unsurprising that PC3 non-PACC parental cells boast a basal high level of anoikis resistance.

While cells in the PACC state have been evaluated in both primary and metastatic tumors in patient samples, presence of PACCs in circulation as CTCs has not been previously shown, likely due to the exclusion of large-nucleated cells by most automated IF-based CTC algorithms. Here, we present anecdotal imaging evidence of large, epithelial-derived cells containing increased genomic content and increased VIM content in the blood of a patient with metastatic, castrate-resistant prostate cancer. Notably, these cells are not of hematopoietic origin, and thus are not megakaryocytes (known to be multi-nucleated). This anecdotal imaging evidence (in conjunction with our deformability and anoikis-resistance data) indicate cells in the PACC state are likely able to survive the circulation during the metastatic cascade. One current limitation of this image analysis approach is the lack of a simple and broadly applicable definition of a cell in the PACC state. In future work, we plan to identify PACC-specific protein signatures that allow for more sensitive and specific “calling” of PACCs in images of cells sourced from in vivo settings.

Another obvious limitation of this study is the lack of in vivo modeling. To make definitive conclusions regarding the survivability and colonization of circulating and disseminated PACCs, in vivo metastasis models must be utilized. In future work, we plan to subcutaneously inject immunocompromised mice with distinct non-PACC parental cells or PACC state populations and quantify several metastasis-relevant parameters including: (1) Differential number of CTCs identifiable in the blood, (2) Differential number of DTCS identifiable in the bone marrow, (3) whether identified CTCs and DTCs are in the PACC state, and (4) Differential number of distant macrometastatic lesions following removal of subcutaneous tumor.

In conclusion, identification of the rare subpopulation of metastasis competent cells remains critically important. Several clinical and experimental observations suggest cells in the PACC state may play an integral role in metastatic disease. In this work, we show that in vitro*,* cells in the PACC state demonstrate increased motility, environment-sensing, and deformability mediated by increased vimentin expression, as well as maintained anoikis-resistance. These characteristics support that cells in the PACC state have increased metastatic potential and suggest the PACC state as a critical phenotype of true metastatic-competency.

## Methods and materials

### Cell culture

Cell culture experiments were performed with the PC3-luc prostate cancer cell line [94]. All cells were cultured with RPMI 1640 media with L-glutamine and phenol red additives. (Gibco) supplemented with 10% Premium Grade Fetal Bovine Serum (Avantor Seradigm) and 1% 5000 U/mL Penicillin–Streptomycin antibiotic (Gibco), at 37 degrees Celsius and in 5% CO2. PC3 cells were authenticated and tested for *mycoplasma* biannually (Genetica). TryplE Express Enzyme without phenol red additive was routinely used as a dissociation reagent unless otherwise stated (Gibco), and all centrifugations were performed at 1000 xg for 5 min unless otherwise stated.

### PACC induction

Cells were plated at a density of 625,000 cells per T75 flask. 24 h after plating, cells were treated with 6 μM (GI50) of the chemotherapeutic drug cisplatin (resuspended in PBS with 140 mM NaCl) for 72 h, unless otherwise indicated (Millipore Sigma). After 72 h of treatment, drug-containing media was removed and replaced with fresh media. Treated cells were kept in culture for up to an additional 10 days, at which point they are considered definitive PACCs. Fresh media was replenished every 4 days. Images of cells undergoing a PAT following the PACC-induction protocol were taken from day 0 through day 15 using the Incuctye S3 Live Cell Analysis System (Sartoris) with daily media changes following removal of treatment.

To ensure purity of the PACC state population for bulk-cell experiments (Western Blot, RT-qPCR), any non-PACC cells remaining in culture following chemotherapeutic PAT induction are excluded via size-based filtration. Treated cells are dissociated from the flask, resuspended in 25 mLs media, and flowed through 15 micron filters (PluriSelect) using back-pressure from an attached 5 mL luer-lock syringe (BD) (5 mL cell suspension/per filter). Cells caught in the filters are then collected in 10 mL media. Collected cells are spun down at 1000 ×g for 5 min and can be reused/replated in any capacity.

### Flow cytometry

Flow cytometry using FxCycle Violet stain (Invitrogen) for analysis of DNA content was performed on 1,000,000 non treated cells and 1,000,000 treated cells immediately following release from chemotherapeutic treatment. The stain was applied according to the manufacturer’s protocol and analyzed using the Attune NxT Flow Cytometer (Thermo Fisher). Live-cell and doublet-exclusion gating was performed unstained controls. Analysis was performed using FlowJo.

### Immunofluorescence

Treated or untreated cells were plated on glass-bottom chamber slides (Falcon, Corning) at various days (1, 5, or 10 days) following release of chemotherapeutic treatment and allowed to adhere overnight. Cells were then fixed with cold 4% methanol-free PFA (Thermo Scientific), for 15 min at room temperature and then washed 3 times for 5 min each with pH 7.4 Phosphate Buffered Saline (PBS) (Gibco). Cells were then simultaneously permeabilized and blocked with a solution of 0.25% Surfact-Amps X-100 (Thermo Scientific) in PBS and 5% Normal Goat Serum (Abcam) in PBS for 60 min at room temperature. Cells were then incubated in primary antibody: Vimentin (D21H3) XP Rabbit mAb (Cell Signaling Technologies) diluted 1:200, and/or Beta-Actin (AC-14) Mouse mAb (Sigma Aldrich) diluted 1:500, and/or Tubulin (YL1/2) Rat mAb (Abcam) diluted 1:200 in a solution of 1% Bovine Serum Albumin (BSA) Fraction V (Fisher Scientific) and 0.25% Surfact-Amps X-100 in PBS overnight at 4 degrees Celsius. Cells were washed 3 times for 5 min each with PBS and incubated in secondary antibody: Goat anti Rabbit IgG H + L Cross-Absorbed Alexa Fluor 555 (Invitrogen) and/or Goat anti Mouse IgG1 H + L Cross-Absorbed Alexa Fluor 488 (Invitrogen) and/or Goat anti Rat IgG H + L Cross-Absorbed, Alexa Fluor 647 (Invitrogen) diluted 1:200 in a solution of 1% BSA and 0.25% Surfact-Amps X-100 in PBS for 3 h at room temperature. Cells were then washed 3 times for 5 min each with PBS and mounted using ProLong Diamond anti-fade mountant with DAPI (Invitrogen) and allowed to cure overnight. All slides were imaged using an AxioCam Mrm (Zeiss) camera on an Observer.Z1 microscope (Zeiss) using the XCite 120Q fluorescence illuminator (Excelitas) and analyzed using ImageJ image analysis software.

### Migration assays

Chemotactic gradients were established using the μ-Slide Chemotaxis system (Ibidi) following the manufacturer’s protocols. Non-PACC parental cells were seeded at 3,000,000 cells per mL of media. PACCs were seeded at 500,000 cells per mL. Positive control cells were seeded in 10% FBS-containing media, were allowed to adhere overnight, were rinsed twice with PBS before uniform solution of 20% FBS-containing media was applied across the μ-Slide. Negative control cells were seeded in serum-free media, were allowed to adhere overnight, were rinsed twice with PBS before uniform solution of serum-free media was applied across the μ-Slide. For the gradient experiment, cells were seeded in serum-free media, were allowed to adhere overnight, were rinsed twice with PBS before a 0–20% chemotactic gradient of FBS-containing media was applied across the μ-Slide. Immediately after addition of the gradient, the cells were imaged via live-cell time lapse microscopy using the EVOS FL Auto Imaging System (Life Technologies). Images were taken with a 10X objective every 30 min for 24 h. Environment chamber conditions were 37 degrees Celsius, 5% CO2, and 20% O2. Immediately following time lapse microscopy, cells were processed for Immunofluorescent labeling of vimentin (see above). In experiments involving vimentin ablation with acrylamide (Sigma), cells were pre-treated with 3 mM acrylamide in complete media for 24 h before beginning a 24-h time lapse.

Time lapse images were analyzed using the Manual Tracking and Chemotaxis and Migration macros in ImageJ image analysis software. All cells analyzed were randomly selected. Cells that underwent division, apoptosis, or moved out of frame were excluded from analysis. 2D surface area measurements of all cells analyzed were also obtained via ImageJ.

### Spontaneous and forced bead motions with magnetic twisting cytometry

Cells were seeded in 96-well stripwell microplates (Corning). Non-PACC parental cells were plated at 30,000 cells/well and PACCs were plated at plated at 3,000 cells/well to account for differences in cell size. RGD-coated ferrimagnetic microbeads (~ 4.5 μm in diameter) were added, anchoring to the cytoskeleton via cell surface integrin receptors of adherent living cells. Spontaneous nanoscale displacement of individual microbeads (~ 100 beads per field of view) was recorded at a frequency of 12 frames/s for t_max_ ~ 300 s via a CCD camera (Orca II-ER, Hamamatsu). Bead trajectories in two dimensions were then characterized by computing mean squared displacement of all beads as a function of time [MSD(t)] (nm^2^) as previously described [95]. We then applied forced motions of the functionalized microbeads using MTC as previously described [96] to measure the stiffness of individual cells. The RGD-coated ferrimagnetic microbeads were magnetized parallel to the cell plating (1,000 Gauss pulse) and twisted in a vertically aligned homogenous magnetic field (20 Gauss) that was varying sinusoidally in time. The sinusoidal twisting magnetic field caused both a rotation and pivoting displacement of the beads that lead to the development of internal stresses that resist its motion. Lateral bead displacement was optically detected with a spatial resolution of 5 nm, and the ratio of specific torque to beads displacement was computed and expressed as the cell stiffness (Pa/nm). The same population of cells (with attached RGD beads) was used to acquire both the cytoskeletal rearrangement and stiffness measurements in the same experiment.

### Atomic force microscopy

Cells were plated in 60 mm dishes. Non-PACC parental cells were seeded at 80,000 cells/dish and PACCs were seeded at 10,000 cells/dish achieve roughly 25% confluency. Cells were incubated overnight to adhere. After 24 h, AFM experiments were done with a cantilever with pointed tips made of Silicon Nitride (SiN). The nominal spring constant of the cantilever was 0.01 N/m (Bruker) on an MFP3D (Asylum Research) instrument. The Cantilever tips were calibrated every time before experiments using thermal fluctuation method [97]. To measure the apical stiffness, cells were indented using contact mode with a maximum peak force of 1 nanoNewton, to get force–displacement curves. All data processing was done using Igor pro software (Wavemetrics). The young’s modulus was obtained by fitting the force–displacement curves with Hertz model, which related the applied force (F) by the cantilever tip to the indentation (δ) and the Young’s modulus (E) using the equation below, where α is the tip opening angle (35°) and v is the Poisson ratio (which is assumed to be 0.5 for soft biological materials) [98].

$$F=\frac{2E \mathrm{tan}\alpha }{\pi (1-{v}^{2})}{\delta }^{2}$$

### Western blot

Treated cells were filtered at various days (1, 5, or 10 days) following release of chemotherapeutic treatment. Each population of filtered cells as well as nonfiltered untreated cells were pelleted and then lysed with an appropriate amount of RIPA Lysis and Extraction Buffer (Thermo Scientific) with Halt Protease and Phosphatase Inhibitor Cocktail (Thermo Scientific) for 30 min, rotating in 4 degrees Celsius. Lysates were spun at 21,000 ×g for 15 min in 4 degrees and supernatant was stored at −80 degrees Celsius. 50 ng of protein (measured by Pierce BCA Protein Assay, following manufacturer’s protocol) (Thermo Scientific) was added to a 1:4 mixture of Laemmli Sample Buffer (BioRad) and 2-Mercaptoethanol (BioRad) and ran through a 4–20% Mini-ProTEAN TGX gel (BioRad). The gel was transferred via Trans-Blot SD Semi-Dry Transfer Cell (BioRad) onto a 0.2 micron Nitrocellulose Trans-Blot Turbo Transfer Pack using the 7 min protocol designed for Mixed Molecular Weights. The blot was blocked in Casein Blocking Buffer (Sigma-Aldrich) for 1 h at room temperature with shaking, and then transferred to primary antibody vimentin (D21H3) XP Rabbit mAb (Cell Signaling Technologies) diluted 1:1000 in casein and incubated overnight at 4 degrees Celsius with shaking OR Monoclonal Anti-Beta-Actin mouse antibody (Sigma) diluted 1:5000 in casein and incubated at room temperature for 1 h. The blot was then washed 3 times for 5 min each with pH 7.4 Tris-Buffered Saline (Quality Biological) with 0.1% Tween 20 (Sigma) (TBST) and incubated in secondary antibody IRDye 700CW Goat anti-Rabbit (Li-Cor) OR IRDye 680RD Goat anti-Mouse (Li-Cor) diluted 1:20,000 in Casein for 1 h at room temperature. The blot was then washed 3 times for 5 min with TBST and imaged using the Odyssey Western Blot Imager (Li-Cor). Densitometry analysis of images was performed using ImageJ image analysis software.

### RT-qPCR

Treated cells were filtered at various days (1, 5, or 10 days) following release of chemotherapeutic treatment. Each population of filtered cells as well as nonfiltered untreated cells were lysed using a QIAshredder Kit (Qiagen) following the manufacturer’s protocol. RNA was extracted from lysates using an RNeasy Mini Kit (Qiagen) following the manufacturer’s protocol. RNA was converted to cDNA (1 ug RNA per reaction) using the iScript cDNA Synthesis Kit (Bio-Rad) following the manufacturer’s protocol. RT-qPCR reactions were performed using SsoFast EvaGreen Supermix (Bio-Rad) following the manufacturer’s protocols on the CFX96 Real-Time PCR Detection System (Bio-Rad). Beta-actin was used as the housekeeping control gene. Gene expression was normalized to a housekeeping gene and calculated with the delta-delta Ct method. The following primers were used:

Vimentin Forward: 5′ TGCCGTTGAAGCTGCTAACTA 3′

Vimentin Reverse: 5′ CCAGAGGGAGTGAATCCAGATTA 3′

Actin Forward: 5′ ACGTGGACATCCGCAAAGAC 3′

Actin Reverse: 5′ CAAGAAAGGGTGTAACGCAACTA 3′

### mRNA nanostring

Cells were treated with either 6 μM cisplatin, 5 nM Docetaxel, or 25 nM Etoposide. Treated cells were filtered 1 day following release of chemotherapeutic treatment. Each population of filtered cells as well as nonfiltered untreated cells were lysed using a QIAshredder Kit (Qiagen) following the manufacturer’s protocol. RNA was extracted from lysates using an RNeasy Mini Kit (Qiagen) following the manufacturer’s protocol. RNA was converted to cDNA using the iScript cDNA Synthesis Kit (Bio-Rad) following the manufacturer’s protocol. The complete product was used as input for hybridization with 770 nCounter PanCancer Progression probes for 16 h according to manufacturer’s protocols. Loaded cartridges were run on an nCounter Sprint (NanoString Technologies). Gene expression data quality control was analyzed using nSolver Analysis Software 4.0.70 (NanoString Technologies). All samples were normalized to the total counts of the nCounter-defined positive controls to reduce lane-to-lane variation from cartridge loading and normalize binding affinity across all samples surveyed. mRNA transcript reads of less than 40 were considered undetected.

### Invasion chambers

The Polydimethylsiloxane (PDMS)-based microfluidic device was fabricated as previously described [99, 100]. The PDMS-based microfluidic devices contained a series of parallel microchannels with varying widths of 3, 6, 10, 20, and 50-µm, lengths of 200 µm, and heights of 10 µm. The microchannels were perpendicular to a 2D cell seeding area and were coated with 20 µg/ml of collagen type I at 37 °C for 60 to 80 min. Cells were dissociated with PBS-Based Enzyme Free Cell Dissociation Buffer (Gibco). 1,000,000 cells per mL of media were loaded into the microfluidic device. Cells were imaged via live-cell time lapse microscopy using the EVOS FL Auto Imaging System (Life Technologies). Images were taken with a 10X objective every 30 min for 24 h. Environment chamber conditions were 37 degrees Celsius, 5% CO2, and 20% O2. 4

### Anoikis-resistance assay

25,000 treated and filtered cells or 25,000 untreated cells were simultaneously plated in (i) a 12-well low-adhesion tissue culture plate (Corning) and (ii) a 12-well normal-adhesion positive control tissue culture plate. After 72 h, both the treated and untreated cells initially plated in the low-adhesion plates were independently transferred to fresh normal adhesion plates, in which they were cultured for an additional 48 h. Both the treated and untreated cells initially plated in normal-adhesion plates were cultured typically for a total of 120 h. 120 h after initial seeding, the cellular viability was measured as a proxy for cellular number or density using the alamarBlue Cell Viability Agent (Invitrogen) according to the manufacturer’s protocols. A 2-h incubation was used and fluorescence (excitation 560, emission 590O was measured via FLUOstar Omega plate reader (BMG Labtech). Anoikis resistance for each condition was calculated by creating a ratio of the viability of the low-adhesion plate challenged cells to the positive control normal-adhesion plated cells for cells from each treatment condition. Alternatively, 120 h after initial seeding, the cellular density was measured using a crystal violet DNA stain, in which cells were fixed with 4% PFA for 15 min at room temperature, stained with 0.05% crystal violet suspended in 20% methanol for 20 min, and thoroughly washed with PBS. After drying, 10% Acetic Acid was used to resuspend the stain. Absorbance (596 nm) was measured using a FLUOstar Omega plate read (BMG Labtech). Anoikis resistance for each condition was calculated by creating a ratio of the absorbance of the low-adhesion plate challenged cells to the positive control normal-adhesion plated cells for cells from each treatment condition.

### CTC detection

Blood was collected from a patient diagnosed with de novo metastatic prostate cancer following castration resistance as previously described in [101]. Blood cells were plated on cell-adhesive (Marienfeld) slides and underwent immunofluorescent stained for a mixture anti-human cytokeratins 1, 4, 5 ,6, 8, 10, 13, 18 and 19, CD45, vimentin, and DAPI as previously described in [101]. Slides were imaged and analyzed as previously described in [101].

### Statistics

Prism9 was used to generate all graphics. Nonparametric T-Tests (Mann–Whitney) were performed using Prism9 to generate all reported P values unless otherwise stated. Elsewhere, nonparametric one-way ANOVA (Kruskal Wallace) was performed using Prism9 to analyze RT-qpCR-generated data, and Rayleigh’s Tests was performed using ImageJ to analyze chemotaxis data. Throughout, an alpha value of 0.05 was used.

NS = nonsignificant = P > 0.05.

* = P < 0.05.

** = P < 0.01.

*** = P < 0.001.

**** = P < 0.0001.

## Supplementary Information

Below is the link to the electronic supplementary material.

Online Resource 1 Video of non-PACC parental cell motility created from 10X time lapse images taken at 30-minute intervals for 24 hours. Supplementary file1 (WMV 4423 KB)

Online Resource 2 Video of PACC state motility created from 10X time lapse images taken at 30-minute intervals for 24 hours. Supplementary file2 (WMV 2801 KB)

Online Resource 3 Video of functional deformability of cells in the PACC state through a 10 μm invasion channel, in response to a 0-20% FBS gradient created from 10X time lapse images taken at 30-minute intervals for 48 hours. Supplementary file3 (WMV 10285 KB) Acrylamide depolymerizes vimentin networks: a) 3 mM acrylamide for 24 hours collapses vimentin networks in PC3 PACC10s without causing cell death. b) 3 mM acrylamide treated for 24 hours collapses vimentin networks, but has no appreciable effect on tubulin or actin polymerization.

Online Resource 4 Video of functional deformability of cells in the PACC state through a 20 μm invasion channel, in response to a 0-20% FBS gradient created from 10X time lapse images taken at 30-minute intervals for 48 hours. Supplementary file4 (WMV 8417 KB) PACC vimentin content does not sufficiently explain PACC state motility: a-f) Linear regression comparing the Integrated Density of VIM signal by immunofluorescence imaging to either the Euclidean distance travelled, directness of motility, or accumulated distance travelled by cells in the PACC state. Linear regression comparing the Mean Fluorescence Intensity of VIM signal by immunofluorescence imaging to either the Euclidean distance travelled, directness of motility, or accumulated distance travelled by cells in the PACC state.
